# Environmentally favorable magnesium phosphate anti-corrosive coating on carbon steel and protective mechanisms

**DOI:** 10.1038/s41598-020-79613-3

**Published:** 2021-01-08

**Authors:** Siyi Yin, Haiyan Yang, Yinghao Dong, Chengju Qu, Jinghui Liu, Tailin Guo, Ke Duan

**Affiliations:** 1 School of Materials Science and Engineering, Southwest Jiaotong Univesity, Chengdu, 610031 Sichuan China; 2 Sichuan Provincial Laboratory of Orthopaedic Engineering, Department of Bone and Joint Surgery, Affiliated Hospital of Southwest Medical University, Luzhou, 646000 Sichuan China

**Keywords:** Civil engineering, Ceramics

## Abstract

Polymer coatings are commonly used to protect carbon steels from corrosion but they are susceptible to weathering and many of them have environmental concerns. Therefore, we studied the possibility of an environmentally favorable inorganic magnesium phosphate cement (MPC) coating for protecting mild steel. A formulation suitable for coating steel was developed by compositional modification [i.e., incremental replacement of dead-burned magnesia (MgO) with magnesium hydroxide (Mg(OH)_2_)] to a road-repair MPC. This modification yielded an acceptable working time and prevented pore formation at the coating-steel interface. Corrosion monitoring by linear polarization and electrochemical impedance spectroscopy for 14 days found that, the MPC coating substantially increased the linear polarization resistance (R_p_) [e.g., day 1: (8.2 ± 1.7) × 10^3^ (nadir value) vs. 495 ± 55 Ω cm^−2^] and charge transfer resistance (R_ct_) (e.g., day 1: 9.3 × 10^3^ vs. 3.8 × 10^2^ Ω cm^−2^). The coated steel underwent neutral sodium chloride (NaCl) salt spray for 2400 h without visible rusting. Immersion for 24 h in liquids simulating the pore fluid indicated that, passivation by the excess MgO in the coating was a major contributor to its anti-corrosive property. Tafel polarization in the liquids found that, corrosion current density (I_corr_) followed the rank: 3.5% NaCl solution (6.0 µA cm^−2^) > 3.5% NaCl solution containing MgO (3.6 µA cm^−2^) > 3.5% NaCl solution containing fragmented MPC (1.7 µA cm^−2^), suggesting that a physical barrier effect and dissolved phosphate ions improved its protection. This study shows that, MPC coating is a promising durable and environmentally favorable anti-corrosive material for protecting steel structures in some applications.

## Introduction

Corrosion of metals and alloys, primarily carbon steels, is estimated to cost 3.4% of the annual gross domestic product of the world^1^. Barrier-type polymer coatings are commonly used to protect steel parts and facilities from corrosion. However, organic coatings are susceptible to weathering and degradation (e.g., sunlight UV, ozone, sandstorms); as a result, their protective ability deteriorates gradually. Furthermore, derived from petroleum and containing organic solvents, many polymer coatings involve environmental issues such as the release of volatile organic compounds (VOCs) and lack of sustainability^2,3^.

With generally complementary physicochemical characteristics to polymers, some inorganic materials have also proved effective in protecting steels. For example, zinc silicate coating has been applied on steel structures (e.g., pipelines, marine facilities) to provide durable protection^3^. However, the curing of this coating is relatively slow and sensitive to environmental conditions (e.g., temperature, humidity). Additionally, because of its shrinkage during curing, it is difficult to build a thick coating without cracking. Furthermore, zinc is increasingly recognized as a contaminant to the soil and ocean^4^. These disadvantages have limited its applications.

Magnesium phosphate cement (MPC) is a family of inorganic binders that use the acid–base reaction between an acidic phosphate salt [typically potassium or ammonium dihydrogen phosphate (KH_2_PO_4_ or NH_4_H_2_PO_4_) and dead-burned magnesia (MgO) to form a binding phase, struvite (MMgPO_4_.6H_2_O; M = K, NH_4_)^5–7^. The reaction causes the reactants to set rapidly (< 40 min) and harden into a strong solid, enabling its use as a fast-setting cement^8,9^. MPC mortars have been used for rapid repair of road surfaces and concrete structures^10–12^. Recent studies explored the use of MPC under corrosive and fire accident conditions^13,14^. Therefore, MPC appears a possible coating material for protecting steel structures working in harsh environments. Several studies prepared magnesium phosphate coatings on carbon steels by phosphating or cathodic deposition, and found the coatings to increase the corrosion resistance^15–17^. However, requiring immersion in an electrolyte, these methods are difficult for coating large structures or preparation of thick coatings. Nevertheless, their results suggest that, magnesium phosphate coatings prepared by other routes (e.g., spraying or spreading an MPC slurry) may also be effective anti-corrosive barriers. Furthermore, coating with an MPC slurry may be readily suitable for large structures and building of thick coatings^15–17^. Furthermore, magnesia is abundantly produced from seawater, and struvite has been used as a slow-release fertilizer for the soil. Consequently, compared with petroleum-derived polymer coatings, MPC is a more sustainable and environmentally favorable material. In fact, because of its binding ability and safety to the human body, MPC has been developed as bone adhesives and fillers for orthopaedic and dental surgeries^18,19^. However, no study has reported the anti-corrosive properties of MPC coating or its possible mechanisms.

The present study reports the development of an MPC material suitable for coating applications and characterization of its anti-corrosive performance and mechanisms.

## Materials and methods

### MPC formulation and coating preparation

A mild steel plate (thickness: 2 mm; C: 0.16%, Mn: 0.47%, Si: 0.28%, S: 0.03%, P: 0.04%, Fe: balance; Hongde Metals, Chengdu, Sichuan, China) was cut into panels 15 × 15 mm or 150 × 70 mm in size. The panels were degreased by sonication in acetone, roughened by grit-blasting with alumina grits, and cleaned with a compressed-air jet.

MPC coating slurries were formulated by modification from a mortar previously developed for road repair, which is described by: dead-burned MgO/KH_2_PO_4_ = 5 (molar ratio), alumina powder (filler)/MgO = 1, borax/(MgO + KH_2_PO_4_) = 0.02, and water/solid = 0.23 (all others: mass ratios). The dead-burned magnesia powder (Chuannai Refractory Plant, Deyang, Sichuan, China) was characterized by D_10_ = 1.7 µm, D_50_ = 11.2 µm, and D_90_ = 40.8 µm. Coating slurries were formulated by incremental replacement of the dead-burned magnesia with magnesium hydroxide [Mg(OH)_2_] (reagent grade, Kelong Chemical, Chengdu, Sichuan, China) while keeping other ratios constant. The slurry was manually mixed with a spatula for 10 min and then spread on panels. A customized fixature was used to control the coating thickness to ~ 500 µm. This thickness was selected because the manual preparation of considerably thinner coatings tended to create surface irregularities such as inconsistency in local thickness. Nevertheless, in future studies, thinner but uniform coatings are expected to be prepared by spraying with specialized equipment. The coatings were allowed to harden at room temperature and characterized as follows.

### Characterizations

The initial setting time of the slurry was measured by the Gilmore needle test. A needle (113.4 g, Φ2.12 mm) was periodically placed on the sample and lifted; when no indent was visible, the time elapsed from the starting of slurry mixing was recorded as the time of initial setting (i.e., loss of flowability). Surface morphology and phase composition were studied by scanning electron microscopy (SEM, Quanta 200) and X-ray diffraction (XRD, Panalytical X’Pert Pro), respectively.

Corrosion properties were evaluated by the linear polarization resistance (LPR) technique, electrochemical impedance spectroscopy (EIS), and the standard neutral salt spray test. The first two electrochemical tests were conducted in a three-electrode mode using a potentiostat (Interface 1010E, Gamry Instruments, Warminster, PA, USA), with a 15 × 15 mm panel embedded in epoxy resin serving as the working electrode, a platinum foil (20 × 20 mm) as the counter electrode, and a saturated calomel electrode (SCE) as the reference electrode. For LPR, all electrodes were immersed in a 3.5% (w/w) sodium chloride (NaCl) solution, and the open-circuit potential (OCP) was monitored. After resting for ~ 30 min to allow the OCP to stabilize, the electrode potential of the panel was scanned from − 10 mV to + 10 mV (vs. OCP) at 0.167 mV  s^−1^, and the LPR was calculated from the slope of the voltammogram recorded. For EIS, after resting for ~ 1 h, a sinusoidal perturbation (amplitude: 20 mV) in the frequency range of 10^–2^–10^5^ Hz was superimposed over the OCP, and the impedance spectrum was measured. EIS spectra were simulated with ZSimpWin 3.1 software. Neutral salt spray test was conducted with 150 × 70 mm panels according to ASTM B-119 (35 ± 1 °C; panel inclination: 30°; LP/YWX-250 spray chamber, Linpin Instruments, Shanghai, China).

### Analyses of anti-corrosion mechanisms

To study the mechanisms of corrosion protection, immersion tests and Tafel polarization were performed. For the immersion tests, 15 × 15 mm panels were immersed separately in three liquids (see “Analyses of anti-corrosion mechanisms”): (1) 3.5% (w/w) NaCl solution, (2) 3.5% NaCl solution containing 10% (w/w) dead-burned magnesia particles (abbreviated as 3.5%NaCl-MgO), or (3) 3.5% NaCl containing 10% (w/w) of MPC particles prepared by grinding of hardened MPC (abbreviated as 3.5%NaCl-MPC). The panels were frequently examined for surface corrosion. For Tafel polarization, panels were immersed individually in the above liquids for 1 min, and the potential was scanned from − 0.3 V to + 0.3 V (vs. OCP) at 0.5 mV s^−1^. Throughout this study, each measurement included three parallel samples.

## Results

### Coating hardening and formulation selection

**Figure 1:**
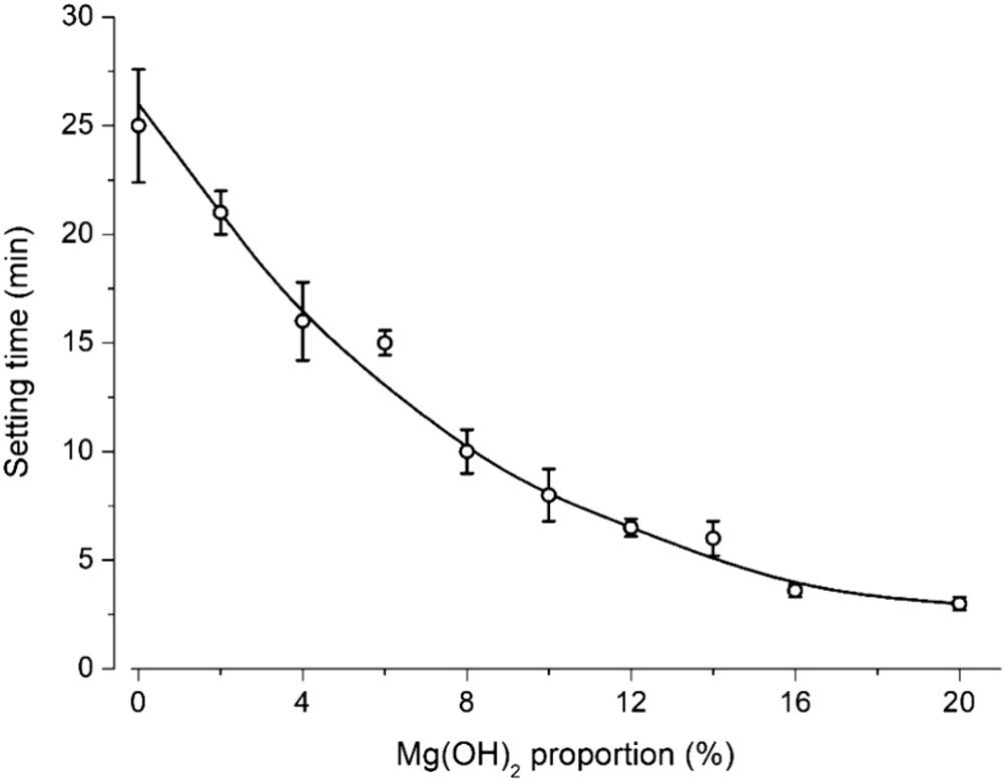
Initial setting times of slurries prepared by incremental replacement of dead-burned magnesia with Mg(OH)_2_.

A series of slurries were tested by incrementally replacing dead-burned magnesia with Mg(OH)_2_ to adjust the workability (initial setting time) while keeping other component ratios constant. When Mg(OH)_2_ replaced 0–8% (w/w) of the dead-burned magnesia, the setting time (Fig. 1) decreased almost linearly, as the uncalcined Mg(OH)_2_ reacted more rapidly (vs. dead-burned magnesia) with KH_2_PO_4_ to form the binder phase. When Mg(OH)_2_ replacement exceeded 8%, the setting time kept decreasing but the slope also decreased gradually, forming a curve.

The coating formulation was selected based on workability of the slurry. Considering several minutes were required for a thorough manual mixing of the components and the mixture became too viscous to handle near the setting time, a setting time of ~ 15 min [i.e., replacement of 6% of dead-burned magnesia] was found to give a reasonable time window (4 min) for mixing and applying the slurry. Therefore, this formulation was selected for subsequent experiments. Nevertheless, when different operation time-windows are required (e.g., different working temperature, equipment), the suitable formulation can also be determined based on the curve (Fig. 1).

### Phase and morphology

**Figure 2:**
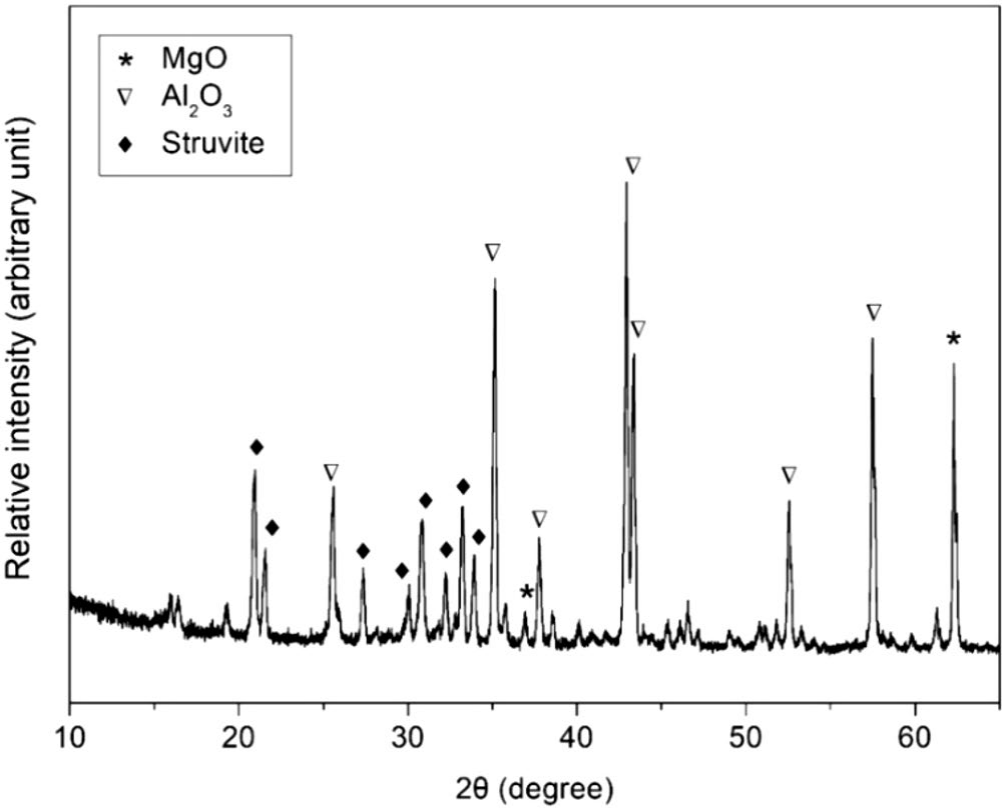
X-ray diffraction spectrum of MPC coating on mild steel.

**Figure 3:**
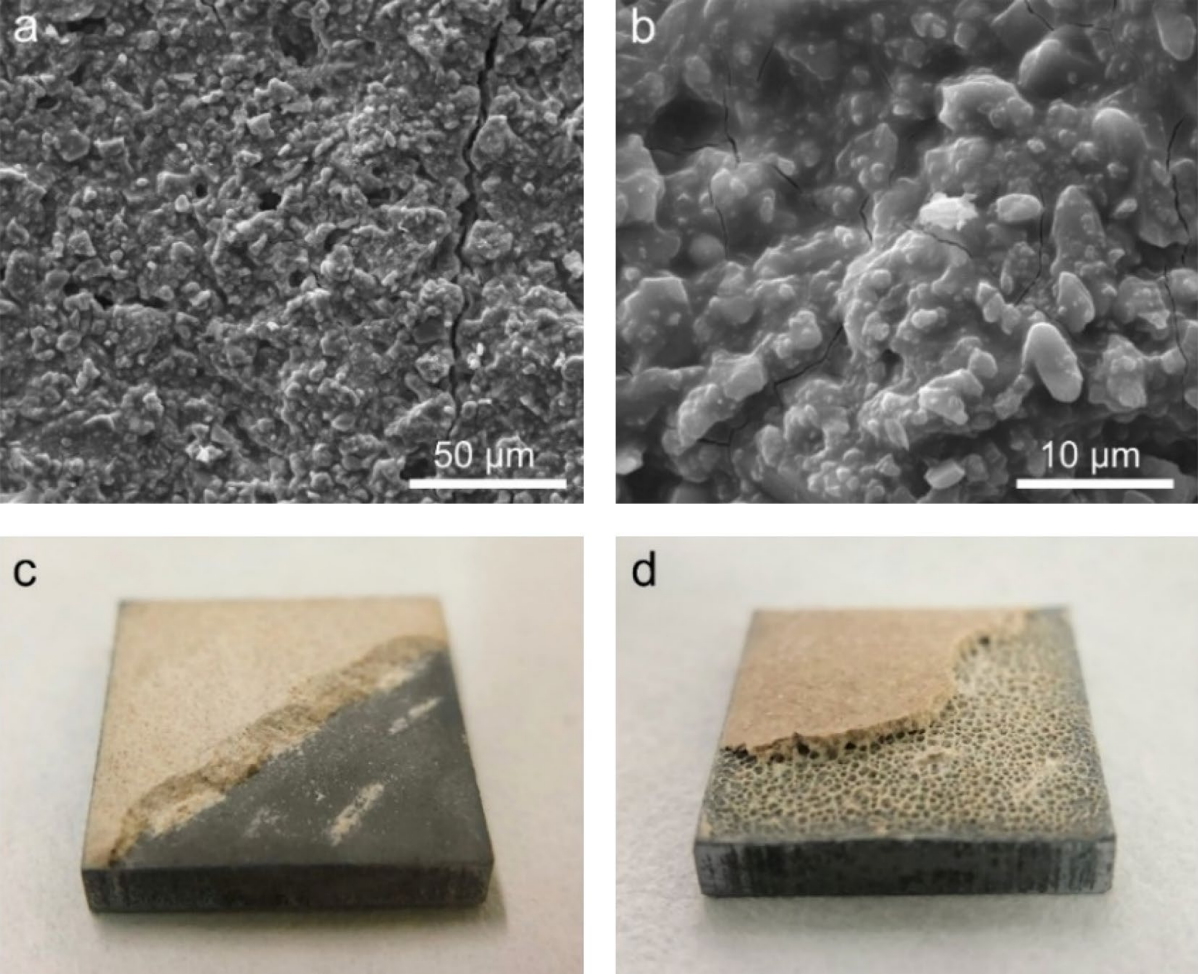
(**a**,**b**) Scanning electron micrographs and (**c**) photograph of an MPC coating [6% of dead-burned magnesia replaced with Mg(OH)_2_)] on a mild steel panel; (**d**) a mild steel panel coated with original mortar [no replacement of dead-burned magnesia with Mg(OH)_2_].

XRD of the coating (Fig. 2) found strong peaks of magnesia and alumina (filler), and moderately strong peaks of struvite [Mg(NH_4_)PO_4_·6H_2_O]. SEM found that (Fig. 3a), the coating had a relatively compact microstructure with some micrometer-sized cracks. Under higher magnifications (Fig. 3b), the coating appeared to consist of particles bound by a matrix. Some coated samples were chiseled with a screw driver to induce brittle fracture-detachment of the coating (Fig. 3c). The cross-section thus exposed showed a visually intimate coating-substrate interface without evident defects (e.g., pores, separation). For comparison, several samples coated with the original unmodified mortar [i.e., 0% replacement of dead-burned magnesia] were also chiseled to expose the interface. In these samples, numerous pores (Fig. 3d) were seen at the coating-steel interface, indicating a defected interface.

### Anti-corrosion properties

#### Linear polarization resistance (LPR)

**Figure 4:**
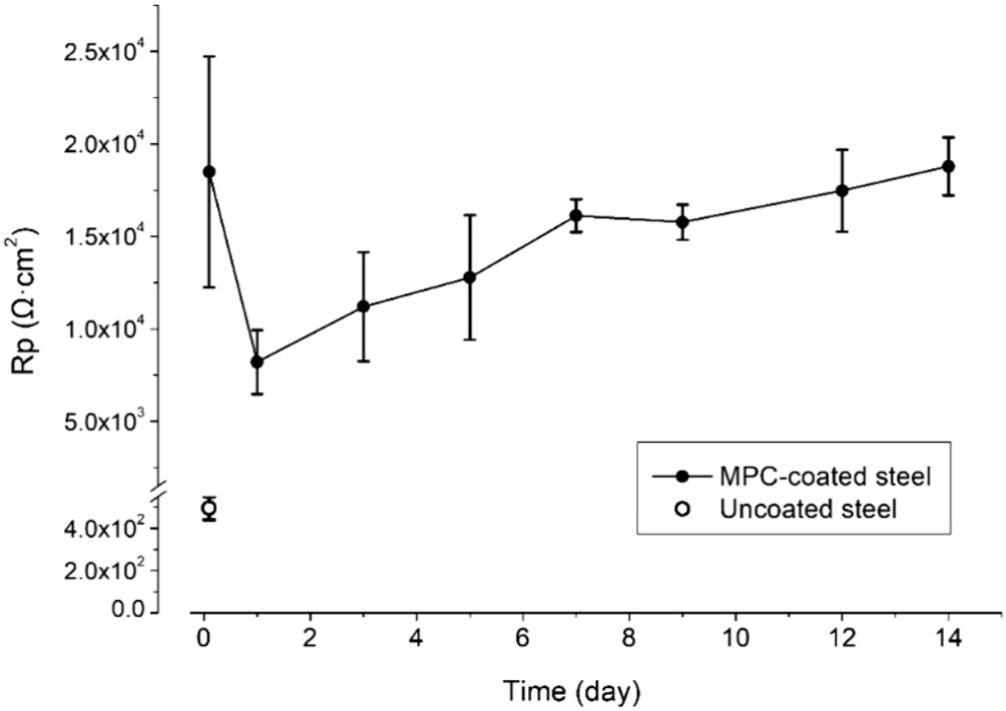
Linear polarization resistances of uncoated and coated mild steel panels during immersion in 3.5% NaCl solution.

LPR (R_p_) is inversely proportional to the corrosion rate of steel in aqueous media^20^. After immersion in 3.5% NaCl for 30 min (Fig. 4), the uncoated steel showed an R_p_ of 495 ± 55 Ω cm^2^. All uncoated panels corroded rapidly in the solution. After ~ 2 h, their surfaces were severely covered by rust and the NaCl solution was severely discolored (not shown). Additionally, the pH of the NaCl solution became increasingly acidic (i.e., hydrolysis of metal ions), preventing LPR monitoring under stable conditions. Therefore, the R_p_ of uncoated steel was not further monitored. In comparison, the MPC-coated panels showed an R_p_ of (1.8 ± 0.6) × 10^4^ Ω cm^2^ after immersion for 30 min, followed by a decrease to a nadir of (8.2 ± 1.7) × 10^3^ Ω cm^2^ at 24 h. Subsequently, it slowly increased till day 14 (end of experiment), reaching (1.9 ± 0.2) × 10^4^ Ω cm^2^. After this 14-day immersion, no rust was visible on any MPC-coated panel.

#### Electrochemical impedance spectroscopy

**Figure 5:**
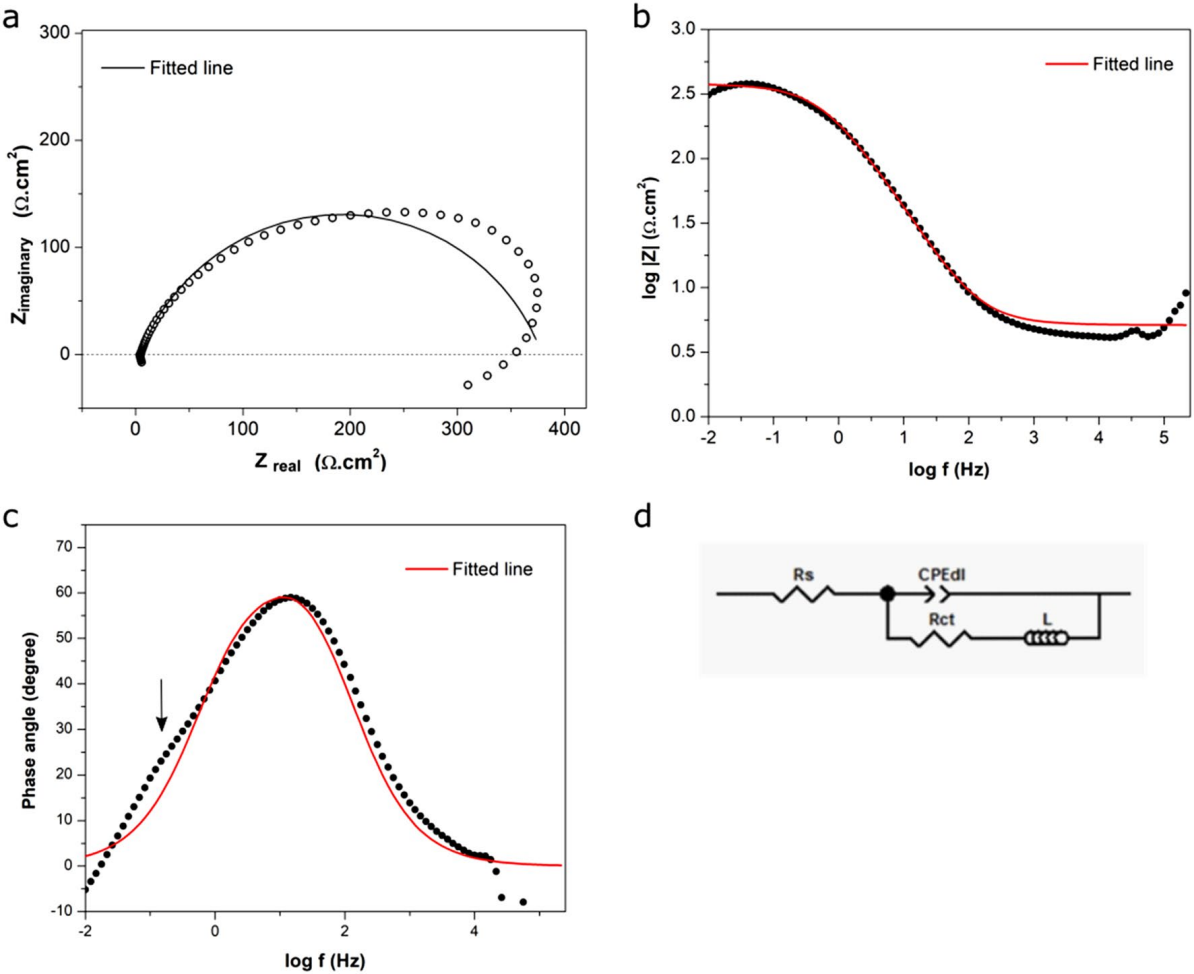
(**a**–**c**) Electrochemical impedance spectrum of uncoated mild steel in 3.5% NaCl solution (**a**: Nyquist plot; **b**,**c**: Bode plots); (**d**) equivalent circuit used to simulate the spectrum; (arrow: shoulder overlapping with major peak).

Figure 5 depicts the EIS spectra of uncoated steel panel immersed in 3.5% NaCl. After 1 h (Fig. 5). The Nyquist plot of the uncoated steel displayed a depressed semicircle at the high- to medium-frequency ranges followed by an inductive loop at the low-frequency end. The semi-circle represents the resistance–capacitance (R–C) characteristics of the steel-solution interface. The inductive loop is attributed to the adsorption of corrosion products (i.e., metal ions) on the electrode surface^21^, as frequently observed from carbon steels undergoing rapid corrosion^22^. The Bode plot (Fig. 5c) exhibited a strong peak of phase angle overlapping with a shoulder (marked by arrow), also indicating the presence of two time constants. Therefore, the spectrum was modeled with an equivalent circuit with two time constants (Fig. 5d), and the fitted parameters are summarized in Table 1. In the circuit, R_s_ represents the solution resistance; CPE is a constant phase element representing the steel-solution interfacial double layer; R_ct_ represents the charge transfer resistance of the double layer; and L models the inductive characteristic of the interface during rapid corrosion. The fitted line deviated noticeably from the measured spectrum (Fig. 5a–c), presumably because of the insufficient accuracy in modeling of the inductive characteristic of the spectrum. The fitted charge transfer resistance (R_ct_) (Table 1), an indicator of the rate of electron transfer at the coating-steel interface (i.e., metal corrosion), was 3.8 × 10^2^ Ω·cm^2^, approximately similar to the R_p_ measured by LPR (Fig. 4). Again, because of rapid corrosion in the solution, the EIS of uncoated steel was not measured after day 1.

**Table 1:** Fitting of EIS spectra of mild steel samples to equivalent circuit.

Time (h)	CPE (S S^n^ cm^−2^)		L (H cm^2^)	R_*ct*_ (Ω cm^2^)
**Uncoated steel**				
1	9.9 × 10^–4^		3.36	3.8 × 10^2^

Time (d)	CPE_1_ (S S^n^ cm^−2^)	R_*po*_ (Ω cm^2^)	CPE_2_ (S S^n^ cm^−2^)	R_*ct*_ (Ω cm^2^)
**MPC-coated steel**				
0.042 (1 h)	3.5 × 10^–4^	4.5 × 10^3^	2.1 × 10^–4^	6.3 × 10^5^
1	1.6 × 10^–8^	2.3 × 10^2^	5.8 × 10^–4^	9.3 × 10^3^
3	6.8 × 10^–7^	5.8 × 10^2^	6.8 × 10^–4^	2.5 × 10^4^
5	9.8 × 10^–7^	7.6 × 10^2^	7.0 × 10^–4^	4.6 × 10^4^
7	1.6 × 10^–6^	1.1 × 10^3^	2.8 × 10^–4^	6.0 × 10^4^
9	1.4 × 10^–6^	1.2 × 10^3^	4.3 × 10^–4^	7.7 × 10^4^
12	6.4 × 10^–6^	1.2 × 10^3^	8.0 × 10^–4^	1.1 × 10^5^
14	1.0 × 10^–6^	1.4 × 10^3^	5.3 × 10^–3^	3.4 × 10^5^

**Figure 6:**
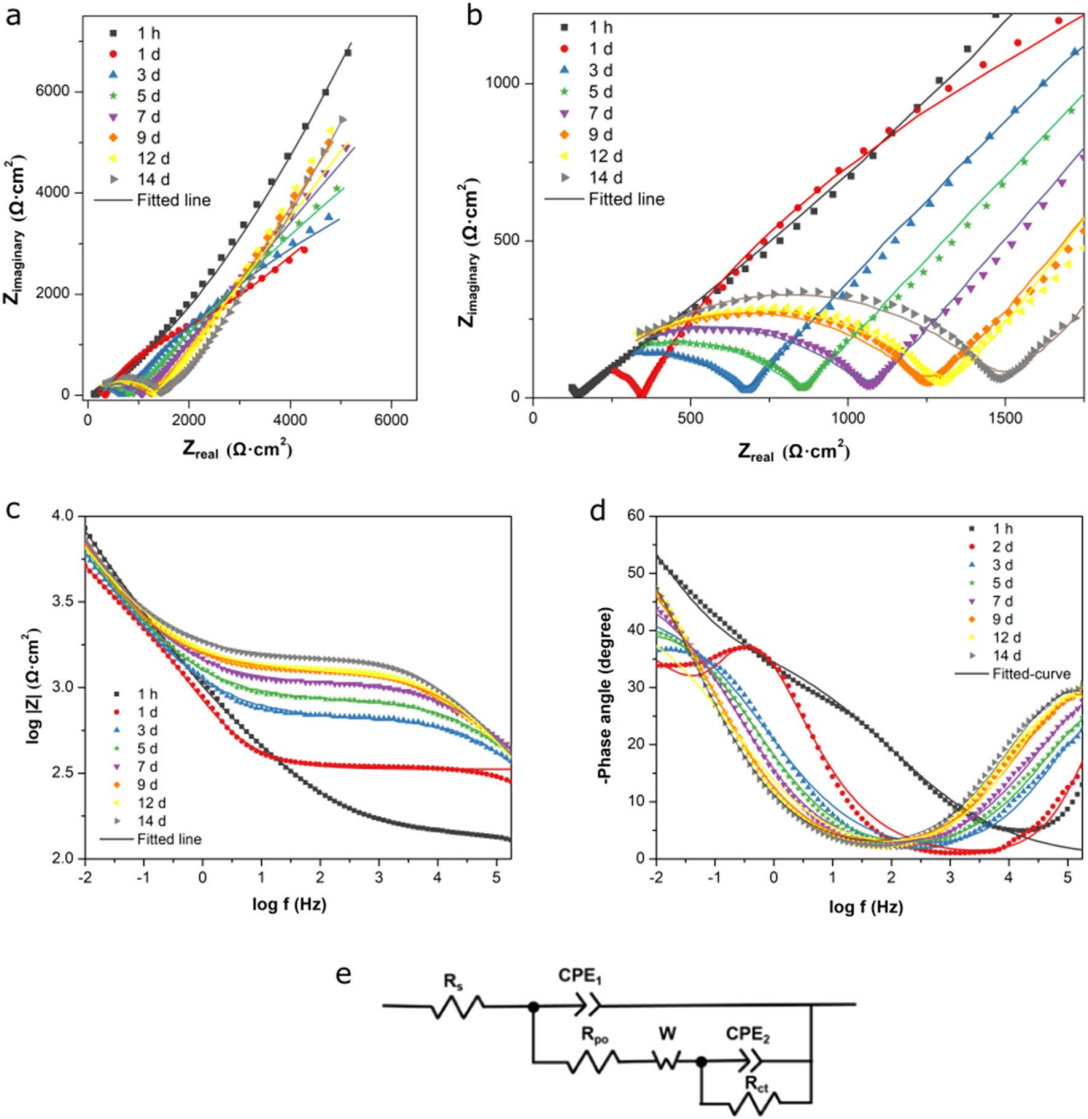
(**a**–**d**) Electrochemical impedance spectra of MPC-coated mild steel immersed in 3.5% NaCl (**a**,**b**: Nyquist plots; **c**,**d**: Bode plots); (**e**) equivalent circuit used to model the spectra.

The Nyquist plot of the MPC-coated panel (Fig. 6a,b) exhibited a depressed semicircle at the high- to medium frequency range followed by upward lines at the low-frequency range, a common pattern from coated metals. With the elapse of time, the semi-circles increased in diameter (Fig. 6b), indicating an increasing resistance to electron transfer (i.e., corrosion) at the steel-liquid interface. The upward lines were approximately 45° in slope, suggesting the presence of a diffusion-controlled process. The Bode plots (Fig. 6d) displayed two peaks of phase angles separated by a valley, indicating two time-constants. Therefore, the spectra were modeled with an equivalent circuit illustrated in Fig. 6c, and the fitted parameters are listed in Table 1. In the circuit, R_s_ represents the resistance of the bulk solution; CPE_1_ is a constant-phase element representing the capacitance of the MPC coating; R_po_ represents the resistance attributed to the pores in the coating; W is a Warbug impedance describing the diffusion-controlled characteristic of the coating; CPE_2_ is a constant-phase element representing the capacitance of the coating-steel interface; R_ct_ represents the charge transfer resistance of the interface. The fitted EIS spectra appeared relatively close to the measured spectra (Fig. 6c,d). The fitted R_ct_ (Table 1) decreased between 1 h and 1 days, and increased subsequently until day 14. Throughout the study, the fitted R_ct_ was at least one order of magnitude higher than that for uncoated steel, indicating a lower corrosion rate. These patterns are consistent with the trend of R_p_ (Fig. 4).

#### Neutral salt spray

**Figure 7:**
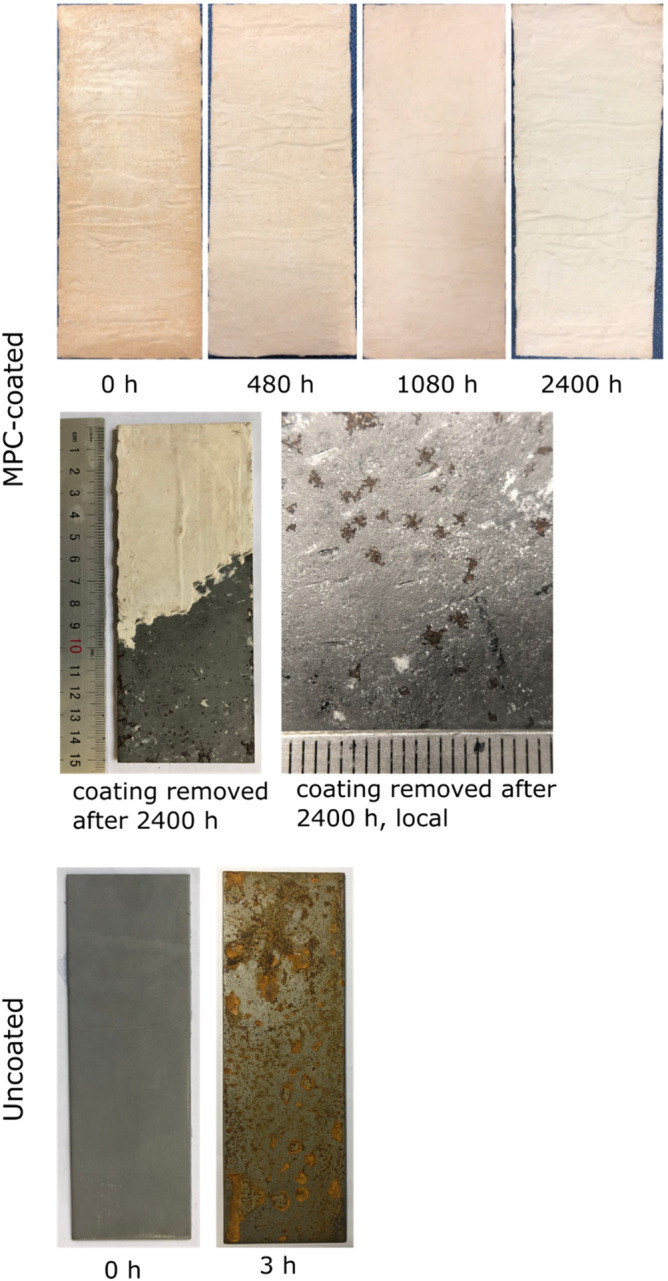
Photographs of a coated mild steel panel (150 × 70 mm) (upper panel) during salt spray for up to 2400 h, (middle panel) the panel chiseled to remove coating after 2400-h salt spray, and (lower panel) an uncoated panel before and after salt spray for 3 h.

After salt spray for 2400 h (Fig. 7), no rust was visible on the coated panels, nor a sign of mechanical degradation of the coating (e.g., cracking, detachment, underlying bulging). During test, the coating appeared progressively whitish. A similar phenomenon (i.e., efflorescence) is frequently observed on hardened ordinate Portland cement. Is attributed to the transport of dissolved ions inside the cement to the surface by water and their subsequent crystallization. The underlying steel was exposed by chiseling off the coating (Fig. 7, middle panel), and the surface was observed to have millimeter-sized corrosion pits. The pits near the edges were larger, probably because the easier diffusion of chloride ions from the edges. In comparison, the uncoated panels rusted rapidly and became substantially covered by rust in several hours. As a result, they had to be removed from the chamber after 3 h.

### Anti-corrosion mechanisms

To probe the mechanisms of corrosion protection by the MPC coating, corrosion of the mild steel in its local medium and the underlying electrochemical processes were studied. Obviously, the steel is exposed to the pore fluid in the coating. The pore fluid can be simulated by suspending powdered MPC in 3.5% NaCl solution. However, considering the MPC contains multiple components that may influence the corrosion rate, it is to necessary to differentiate the contribution of each. Chemically, the hardened MPC comprises magnesia (stoichiometrically excess vs. KH_2_PO_4_), struvite, alumina, and potentially trace (unreacted) KH_2_PO_4_ (Eq. 1; Fig. 1). Magnesia is a mediumly strong base, which, according to the Pourbaix diagram, may passivate the steel surface by forming a hydroxide layer. Struvite and potentially trace KH_2_PO_4_ can liberate phosphate ions, which may form insoluble precipitates on the surface to retard corrosion. The alumina filler, calcined in nature, is commonly known to be unreactive. Therefore, panel corrosion and voltammograms were monitored in three liquids (Sect. 2.3) and compared.

**Figure 8:**
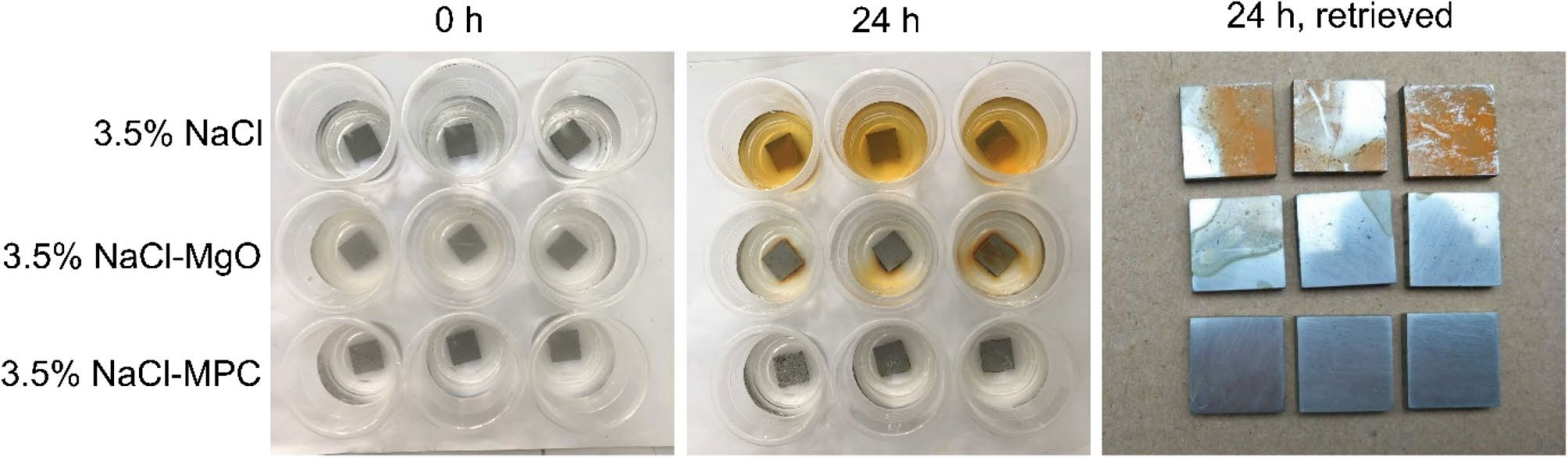
Photographs of uncoated mild steel panels immersed in three liquids for 0 and 24 h.

After immersion in 3.5% NaCl for 24 h, the uncoated panels showed corrosion and the liquid became brown (Fig. 8). In comparison, those immersed in 3.5%NaCl-MgO underwent a more moderate corrosion, whereas those in 3.5%NaCl-MPC had further less corrosion. These indicate that, ions dissolved from magnesia acted as an effective corrosion inhibitor, and those from other phases in the hardened MPC further enhanced its protective ability.

**Figure 9:**
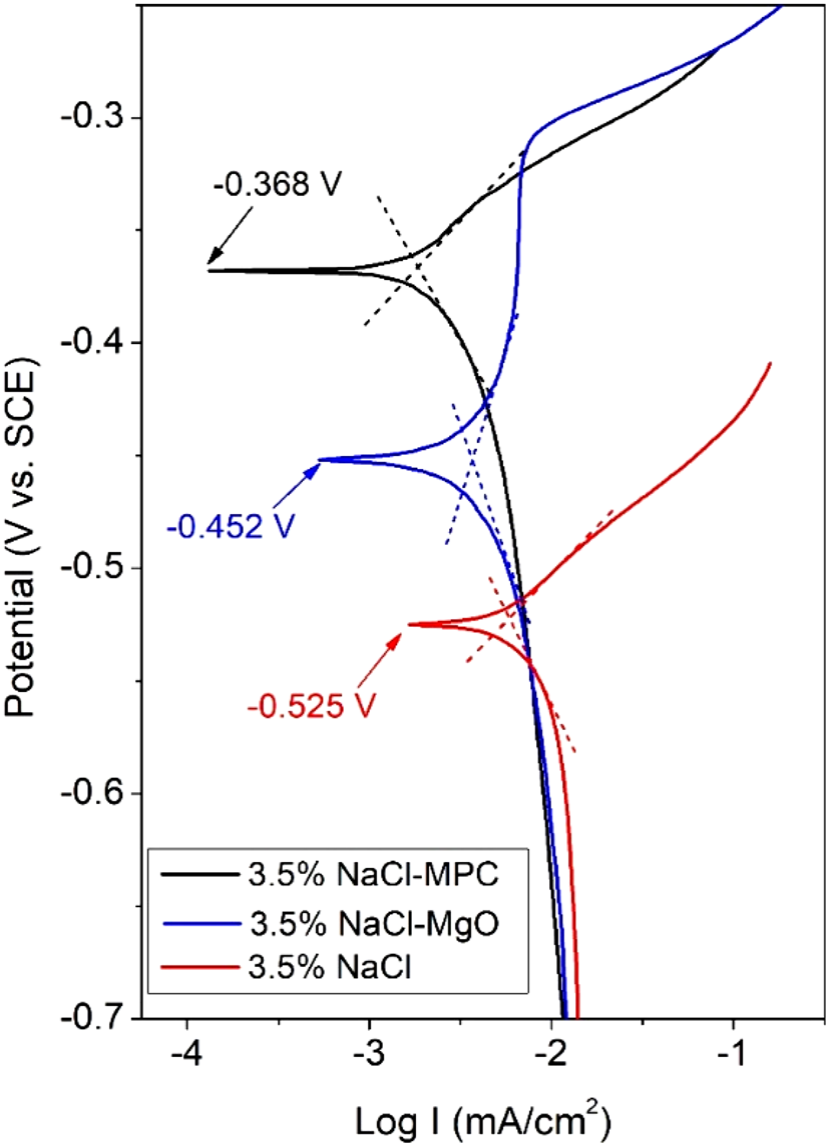
Tafel curves of uncoated mild steel panels immersed in three liquids.

Tafel polarization of the samples in the three liquids found that (Fig. 9), the anodic branches of the voltammograms decreased in the order: 3.5%NaCl > 3.5%NaCl-MgO > 3.5%NaCl-MPC whereas the cathodic branches changed moderately. The corrosion current densities (I_corr_) followed the rank of: 3.5%NaCl (6.0 µA cm^−2^) > 3.5%NaCl-MgO (3.6 µA cm^−2^) > 3.5%NaCl-MPC (1.7 µA cm^−2^). The corrosion potentials (E_corr_) of the panels immersed in 3.5%NaCl-MgO and 3.5%NaCl-MPC shifted sequentially toward the positive direction (vs. 3.5%NaCl) by 73 mV and 157 mV, respectively. According to the mixed potential theory, these changes indicate a progressively suppressed anodic reaction [Fe(0) → Fe^2+^ + 2e]) in 3.5%NaCl-MgO and 3.5%NaCl-MPC relative to 3.5%NaCl.

## Discussion

These results indicate the MPC coating to be an effective anti-corrosive barrier for the mild steel. The coating was formulated by modification from an MPC mortar developed for road repair. Although suitable for fast road repair, the mortar sets too slowly (~ 25 min) for coating applications; more importantly, it is highly acidic (pH ~ 2) shortly after mixing due to the dissolution of KH_2_PO_4_. The acidic slurry reacts rapidly with the steel to form hydrogen gas, creating pores at the coating-substrate interface (Fig. 3d). Therefore, more reactive Mg(OH)_2_ was incrementally introduced to accelerating the slurry hardening and reducing the slurry acidity. Several other methods were also found effective for eliminating interfacial pores, including pre-phosphating of the steel, natural rusting in the air before coating, or partial replacement of KH_2_PO_4_ with K_2_HPO_4_. However, they were found to be inconvenient (pre-phosphating/rusting) or reduce the anti-corrosive capability (replacement with K_2_HPO_4_). Nevertheless, other innovative approaches to formulating MPC coatings may be possible and deserve exploration.

Even when the MPC coating was absent, suspension of MgO or ground MPC particles decelerated the steel corrosion in 3.5%NaCl (Fig. 8), indicating that magnesia and other components dissolved from the MPC coating can inhibit the steel corrosion. Tafel scans (Fig. 9) suggest that they act primarily by inhibiting the anodic reaction. These can be explained by the Pourbaix diagram. A mediumly strong base, magnesia (pH ~ 10.6) may passivate the steel surface by forming a protective iron hydroxide precipitate. This is analogous to the passivation of steel rebars in concrete, in which calcia (pH ~ 14) forms an iron hydroxide precipitate on the steel^23^. Struvite and potentially trace unreacted KH_2_PO_4_ release phosphate ions into the pore fluid, which may combine with Fe^2+^ liberated from sites of corrosion to form an iron phosphate precipitate, covering these sites. Phosphate salts are known effective corrosion inhibitors for carbon steels^24^. This chemical corrosion-inhibiting mechanism is desirable, as when small uncoated areas are present (e.g., defective coating operation) they are, to some degree, protected chemically. In fact, we observed that, when MPC-coated coupons were marked with X-shaped scribes and placed in the salt spray chamber shortly after coating hardening, the scribes became gradually partially filled. After salt spray for 240 h, many scribes were less noticeable (not shown). This reflects the migration of reactive species (e.g., Mg^2+^, PO_4_^3−^) dissolved from the adjacent coating mass and local precipitation in the scribe, analogous to the self-healing of small cracks in concrete when water is available^25^.

The dense morphology of the coating (Fig. 2) and ~ 45° upward diffusion-controlled lines suggest a physical barrier effect. In 3.5% NaCl, R_p_ decreased between 1 h and 1 day, possibly because at 1 h the solution had not sufficiently penetrated the coating. With gradual infiltration of the solution, corrosion increased, and R_p_ reached the nadir on day 1. From day 1 to 14, R_p_ and the diameter of the semicircles in EIS kept increasing, likely because the corrosion product clogged some pores in the coating, impeding the ingression of reactants for corrosion (e.g., O_2_)^26^. Therefore, the combination of physical and chemical inhibition confined corrosion to local points (Fig. 7) and retarded its progression.

These results show that, MPC may be applied as effective anti-corrosive coatings for mild steel. This is expected to be valuable for structures serving under harsh conditions. For entering actual applications, more requirements must be satisfied, such as tolerance of temperature cycles and resistance to acid rain. Inspired by advances in cement technologies, various fillers and industrial wastes (e.g., flyash, mineral fibers) may be added into the coating to create products that are not only effective but also benign to the environment. These will be investigated systematically and reported in the future.

## Conclusion

A MPC slurry suitable for coating mild steel was developed by incremental replacement of dead-burned magnesia in a road-repair mortar with Mg(OH)_2_. This modification yielded an acceptable working time, and reduced pore formation at the coating-steel interface. In 14-day electrochemical test, the coating increased the corrosion resistance of the steel. The coated steel underwent 2400-h salt spray without visible rusting. Immersion in liquids simulating pore fluid indicates magnesia to be a major contributor to its anti-corrosive ability. A barrier effect and dissolved phosphate ions further enhanced its protection. The coating is expected to be a promising durable and environmentally favorable anti-corrosive material for steel structures in some applications.
